# Mitigating disruptions, and scalability of radiation oncology physics work during the COVID‐19 pandemic

**DOI:** 10.1002/acm2.12896

**Published:** 2020-05-20

**Authors:** Arash Darafsheh, Hossein Lavvafi, Reza Taleei, Rao Khan

**Affiliations:** ^1^ Department of Radiation Oncology Washington University School of Medicine St. Louis MO 63110 USA; ^2^ William E. Kahlert Regional Cancer Center Westminster MD 21157 USA; ^3^ Department of Radiation Oncology Sidney Kimmel Medical College at Thomas Jefferson University Philadelphia PA 19107 USA

**Keywords:** contingency plan, coronavirus, COVID‐19, medical physics, pandemic, radiation oncology

## Abstract

**Purpose:**

The COVID‐19 pandemic has led to disorder in work and livelihood of a majority of the modern world. In this work, we review its major impacts on procedures and workflow of clinical physics tasks, and suggest alternate pathways to avoid major disruption or discontinuity of physics tasks in the context of small, medium, and large radiation oncology clinics. We also evaluate scalability of medical physics under the stress of “social distancing”.

**Methods:**

Three models of facilities characterized by the number of clinical physicists, daily patient throughput, and equipment were identified for this purpose. For identical objectives of continuity of clinical operations, with constraints such as social distancing and unavailability of staff due to system strain, however with the possibility of remote operations, the performance of these models was investigated. General clinical tasks requiring on‐site personnel presence or otherwise were evaluated to determine the scalability of the three models at this point in the course of disease spread within their surroundings.

**Results:**

The clinical physics tasks within three models could be divided into two categories. The former, which requires individual presence, include safety‐sensitive radiation delivery, high dose per fraction treatments, brachytherapy procedures, fulfilling state and nuclear regulatory commission's requirements, etc. The latter, which can be handled through remote means, include dose planning, physics plan review and supervision of quality assurance, general troubleshooting, etc.

**Conclusion:**

At the current level of disease in the United States, all three models have sustained major system stress in continuing reduced operation. However, the small clinic model may not perform if either the current level of infections is maintained for long or staff becomes unavailable due to health issues. With abundance, and diversity of innovative resources, medium and large clinic models can sustain further for physics‐related radiotherapy services.

## 1 INTRODUCTION

Coronaviruses belong to a large family of viruses that have been common in humans and many other species. The recent outbreak of the novel COVID‐19 respiratory disease around the world, first detected in Wuhan, China, in 2019, is caused by a new coronavirus, called severe acute respiratory syndrome coronavirus 2 (SARS‐CoV‐2) which is the seventh coronavirus known to infect humans.^1^, ^2^

On 11 March 2020, the World Health Organization (WHO) declared the COVID‐19 outbreak a pandemic.^3^ As of this writing (11 April 2020), over 1.7 million confirmed infections have been reported in 210 countries and territories and two international conveyances around the world; United States with over 500,000 cases has the highest reported infections.^4^

There is currently no specific treatment or vaccine for COVID‐19. Similar to influenza and other contagious respiratory diseases, to prevent illness one should avoid getting exposed to the virus which is spread by respiratory secretions of an infected individual.^5^, ^6^, ^7^ Doremalen et al. experimentally studied the viability of SARS‐CoV‐2 in aerosols on various surfaces, the longest survival of contagion was observed on stainless steel and plastic with 5.6 h and 6.8 h median half‐life, respectively.^8^ The study concluded that aerosol and fomite transmission of SARS‐CoV‐2 is plausible, since the virus can remain viable and infectious in aerosols for hours and on surfaces up to days (depending on the inoculum shed).

As the recommendations on how to avoid the contagion continue to evolve, the US Centers for Disease Control and Prevention (CDC) provides updated general and specific guidance about the current pandemic.^9^ The best approach at this moment is through “social‐distancing” where physical contact is completely avoided in addition to maintaining a distance of about 6 feet (~2 m) between two individuals irrespective of the status of disease.

In the face of rapid COVID‐19 spread, states, and jurisdictions have imposed restrictions on non‐essential contact, and stay‐at‐home or shelter‐in‐place orders. Though, it is helpful in containing the disease and avoiding overwhelming the healthcare infrastructure, it is a serious impediment to radiation oncology physics clinical services. In a report involving 138 hospitalized COVID‐19 patients in Wuhan, it was reported that 41.3% of them were presumably infected in the hospital of which 29% were hospital staff and 12.3% were patients already hospitalized for other reasons.^10^ Since the COVID‐19 infection risk is serious, it is crucial to prevent and control its spread in the radiation oncology departments. It is therefore necessary to set efficient and feasible prevention and control measures. Several radiation oncology centers have reported their experiences and contingency plans to counter the impact of the current pandemic on the clinical workflow.^11^, ^12^, ^13^, ^14^, ^15^, ^16^, ^17^, ^18^, ^19^, ^20^, ^21^, ^22^, ^23^

Filippi et al. from Italy provided a practical guidance for radiation therapy (RT) departments based on prioritization, problem analysis, and suggested solutions. Guidelines were based on the core objective of providing availability of RT to the patients while ensuring safety of the patients, health professionals and caregivers, and on special management of suspected or COVID‐19 positive cancer patients.^11^ This was accomplished by staff re‐organization, reduction of patients’ access to RT visits through hypofractionated regimens and postponing follow‐up visits. Similarly, Achard et al. emphasized the need for hypofractionated regimens, when feasible, to decrease the access of cancer patients to the hospital and limit potential diffusion of COVID‐19.^12^ Braunstein et al. in an effort to mitigate risk to patients and optimize resource utilization has suggested omitting, delaying, or reducing radiotherapy for breast cancer, where appropriate.^24^ Marijnen et al. provided a consensus statement regarding radiotherapy options for rectal cancer during the pandemic.^25^ A practice recommendation for head and neck cancer RT during the pandemic has been provided by Thomson et al.^26^ Yerramilli et al. presented their departmental approach in to triaging and shortening RT for oncologic emergencies at a major comprehensive cancer center in New York City. ^27^

Wu et al., based on their experience in Wuhan, advised on patient and healthcare worker screening, health education, staff training, zoning, and adapting the workflow.^13^ Papachristofilou et al. from Switzerland developed a contingency plan by streamlining human resources through staff preparation, setting priorities, establishing a response team and gathering support, to reassure staff and patients regarding protection during such a calamity.^14^

Krengli et al. from Italy summarized “lessons learned” as: swift action in adaptation of new rules; social distancing; disinfection of rooms and equipment; wearing proper personal protective equipment (PPE); maintaining the stockpile of sanitation tools and PPEs; effective communication with the patients and the personnel; flexibility of the personnel, when needed, in covering different tasks; and sharing experience among different centers.^15^ These were adapted to a varying degree in ongoing pandemic in the United States.^16^

An et al. provided a list of appropriate PPEs needed at different protection levels, which include protective cap, N95 respirator, alcohol‐based disinfectant hand sanitizer (75% ethanol), goggle and face shield, sterile latex gloves, isolation gown, protective clothing, shoe covers and protective boots, surgical mask, and adult diaper.^21^

According to medical physics practice guidelines, the physicists are involved to a varying degree in administration, clinical services, education, informatics, equipment performance evaluation, quality assurance, and safety.^28^, ^29^ Particularly, in a radiation oncology department, medical physicists provide supporting services to radiation oncologists (RO) and clinicians in almost all clinical scenarios. These include, but are not limited to, acceptance and commissioning of new equipment, dosimetry services, planning, patient's chart quality assurance (QA) and patient‐specific QA, radiotherapy documentations, radiation safety, etc. A majority of these tasks require team work, cross‐checking, direct supervising, and in some cases providing consultation and face‐to‐face patient interactions.^30^ In a devastating situation such as the outbreak of an epidemic, wearing different hats can be very useful in mitigating the situation. During the last few weeks of the COVID‐19 outbreak, the level of medical physics tasks has transitioned and evolved in such a short time that has no precedence. Even though medical physics is an integral part of radiotherapy, the emerging literature either completely lacks its prospective, or has not covered it in depth. In this work, we aim to provide an update on how various medical physics services and tasks have been adapted to new realities to accomplish the goal of RT while observing the guidance to protect the staff, patients, and personnel from either falling for the contagion or becoming a carrier for the contagion. Another objective of our work is to review changes in the light of the US healthcare model. To this end, we will provide a comparison of three model facilities with different scales of operations to represent a major subset of medical physics practice across the United States in standing up to this challenge at this point in time. Due to the transitory nature of the COVID‐19, we would caution the reader that long‐term sustainability of these results is not guaranteed.

## 2 MATERIALS AND METHODS

### 2.A Practice stratification

We divided clinical physics practice into three main categories: small, medium, and large. A modest portion of radiation oncology in the United States is practiced in small‐sized clinics with 1–2 full time equivalent (FTE) individuals responsible for the medical physics activities. Compared to a medium or large physics groups, a medical physicist is likely to be responsible for a broad range of duties, and for both implementing and overseeing a series of quality control (QC) tests and audits to ensure patient safety. For example, the small practices face challenges of effective peer‐review due to costs and solitary nature of their environment.^31^ The medium size clinic was defined as having 3‐10 clinical physicists, with 3–6 radiotherapy linear accelerators (linacs), at least one specialized program of MRI‐guided RT (MRIgRT) or a clinical proton therapy, or stereotactic radiosurgery and a clinical medical physics residency program. The large clinic includes more than ten physicists, more than 6 linacs, special treatment programs, MR simulator, MRIgRT and proton therapy, and both medical physics graduate and residency education programs. The scope of services across these three models is significantly different to warrant such a stratification. Table 1 provides a snapshot of a typical spectrum of services practiced under various categories.

**Table 1:** Typical tasks in different settings for three categories of medical physics services in a radiation oncology department.

Small Clinics (1–2 physicists, 1–2 linacs, HDR, 15–25 EBRT per day)	Medium Clinics (3–10 physicists, 3–6 linacs, brachytherapy, specialized RT, 75–150 EBRT per day)	Large Clinics (10+ physicists, multiple linacs, brachytherapy, specialized treatments, 150+ EBRT per day)
Initial plan quality check (EBRT and brachytherapy) Pre‐checks for plans Plan revision and weekly chart check Patient‐specific QA Periodic machine QA *In vivo* dosimetry Physics consult Setup verification Acceptance and commissioning of imaging and therapy devices and accessories	All tasks listed in the left column TBI/ TSET treatments Specialized RT with either, proton, or MRIgRT or radiosurgery Possible coverage of multiple services (e.g. SBRT/SRS, Gamma Knife®/ Tomotherapy/ CyberKnife®, etc.) Clinical physics support for 1‐2 satellite clinics Education (Physics residency, therapist training, RO clinical residency) Clinically focused research Clinical trials	All tasks listed in the middle column Additional physics support staff (Physics assistants, quality assurance staffs) Possible coverage of multiple services (e.g. multi‐room proton therapy, MRIgRT, SBRT/SRS, Gamma Knife®, CyberKnife®) Clinical physics support for over 3 satellite clinics Education (Medical physics graduate and certificate programs) Research and development

EBRT: External beam radiation therapy; MRIgRT: MRI‐guided radiation therapy; SBRT: Stereotactic Body Radiation Therapy; SRS: Stereotactic Radiosurgery; TBI: Total Body Irradiation, TSET: Total Skin Electron Therapy.

### 2.B Level of infections and mortality

**Fig. 1:**
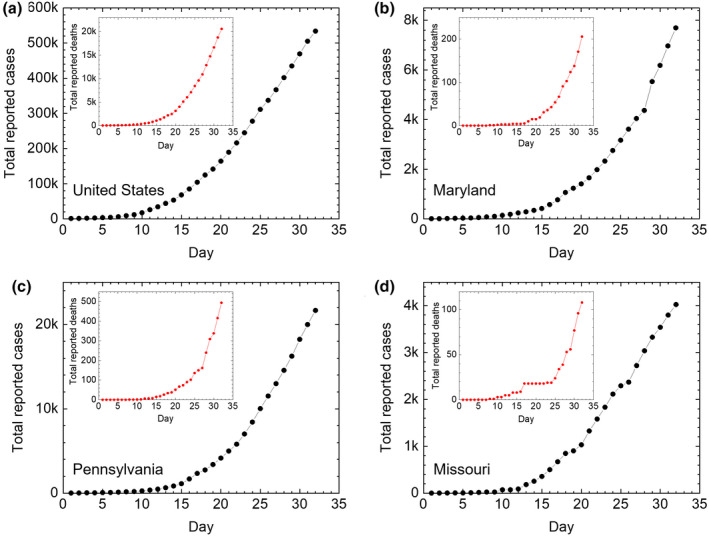
Cumulative reported cases and deaths for COVID‐19 for the entire USA (a), States of Maryland (b), Pennsylvania (c), and Missouri (d) provide contextual data for small, medium and large clinics considered in this paper. The approximate population of each of these states is 9, 12.8, and 6.1 million, respectively. Day 1 in all panels corresponds to 11 March 2020. Data were taken from Ref. 33.

The number of reported cases with COVID‐19 positive, as of this writing, continues to increase. The mortality of infected individuals is also on the rise. Figure 1(a) provides the data of reported cases in the United States, the inset in the figure provides corresponding mortality due to complications from the disease. The plots in Fig. 1(b), Fig. 1(c), and Fig. 1(d) correspond to the location of the small, medium, and large clinics, respectively, considered in this paper. Figure 1 provides an important context to the state of the disease.

### 2.C Optimization of services

**Fig. 2:**
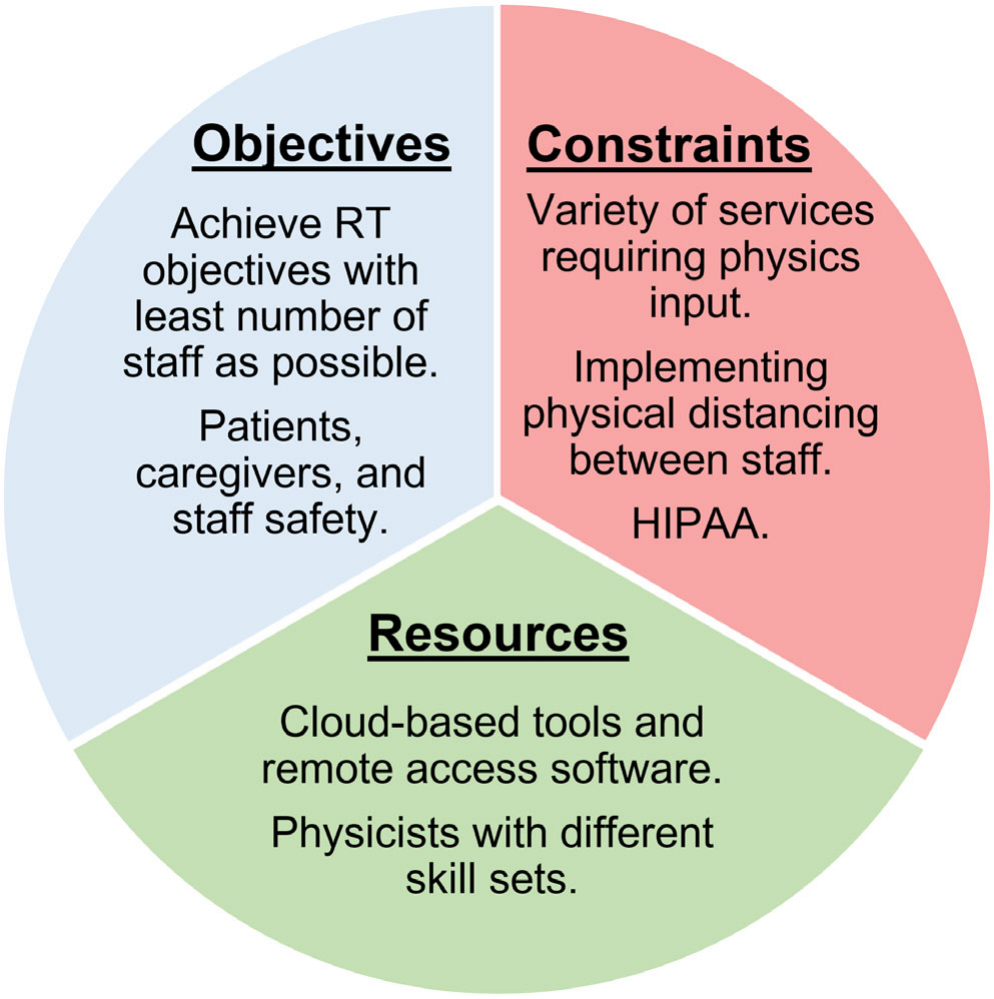
Optimization of resources in pursuit of RT objectives under constraints of “social distancing” and availability of remote services.

The main objective of medical physics practice, for previously stated categories, continues to be safe, efficient, and uninterrupted delivery of RT while maintaining safety of all staff, patients, and caregivers during the disease outbreak. Figure 2 presents an overview of the optimization task with interdependence on constraints and resources at hand.

#### 2.C.1 Constraints

CDC guidelines, local, state and federal regulations; travel restrictions; minimization of personnel traffic in the clinical space, reducing duration of physical presence; physical distancing measures described by “social distancing” while interacting with patients, staff and other members of the public; actual number of trained physicists for a particular clinical task; the information technology (IT) technical support of the clinic; clinical engineering support personnel; dosimetry support; individual office space; number of software and hardware licenses for functioning remotely; virtual private networks (VPN) setup; adherence with data protection and patient confidentiality rules (e.g. Health Insurance Portability and Accountability Act (HIPAA) in the US) for remote hardware and software; network bandwidth, staff training to use these software; sanitizing and cleaning and access to PPE supplies; physical presence requirements for certain special procedures, etc. constitute some of the constraints to operate.

#### 2.C.2 Resources

Availability of remote services in the form of remote desktop connections; virtual environments for treatment management systems and dose planning software; required licenses for these services; HIPAA compliant audio/video, and SMS capable communication devices, such as smart cellular phones and tablet computers; VPNs with digital and network switch based firewalls; bandwidth with dedicated back‐up lines to and from the datacenter to the hospital; city‐wide network bandwidth for consumers; spare computer hardware and mobile computers for working from home‐office; business level video conferencing applications that provide remote meeting and discussion forum tool, such as Zoom (Zoom Video Communications Inc., San Jose, CA), Skype for business (Skype Technologies, Palo Alto, CA), GoToMeeting (LogMeIn, Inc., Boston, MA), Cisco Webex (Cisco Systems, Milpitas, CA), and Microsoft Teams (Microsoft, Redmond, WA); utility of consumer level services, such as WhatsApp Messenger (Facebook, Inc., Menlo Park, CA) and FaceTime (Apple Inc., Cupertino, CA) for professional discussion when no protected health information (PHI) is shared are some of the critical resources which could be made available to aid in disruptive situation.

It is crucial that the resources used to manage remote services are HIPAA compliant per United States 45 CFR (Code of Federal Regulations) Parts 160, 162, and 164 which includes transactions and code set standards, identifier standards, privacy, security, enforcement, and breach notification rules. This will ensure securing the transfer of patient information and avoid any security breaches involving PHI. According to the vendors, Zoom, Skype for business, GoToMeeting, Cisco Webex, and Microsoft Teams are HIPAA compliant when configured correctly and used in a manner compliant with HIPAA rules. However, Apple iMessage service or WhatsApp are not meant to be used for sharing PHI due to lack of appropriate security measures such as end‐to‐end encryption or lack of securely limited controlled access.

## 3 RESULTS

In a small clinic scenario employing two radiation oncologists and two physicists, one dosimetrist, 3–4 radiation therapists, with one linac treating about 20–25 treatments each day and about two gynecology HDR patients weekly, the disruption and mitigation is summarized as follows:

Preparatory meetings involved planning for continued RT operation for upcoming disruptive situations. The staff who could work remotely from home and the minimum number of individuals required for sustained operation in the clinic were identified. Hardware resources such as laptop or personal computer, and IT support for allocation of licenses and firewall management were assessed. Team members were instructed and trained for remote access setup and how to login/logout properly in order to minimize disruptions in the network connection. Establishing and testing of IT authorized remote connection software were performed for all staff, whether working from home or from their offices, in order to conduct all meetings according to the recommended protocol of social distancing. Microsoft Teams software was used to establish communication between dosimetry and physics regarding treatment plan review or evaluating patient‐specific QA requirement for the day. Microsoft Team video chat with screen sharing provided required tool to review treatment plans with the physician, physicist, and dosimetrist together to improve plan quality before electronic review and plan approval.

Video conferencing was also utilized for chart rounds, daily huddle, QA meetings which allowed all professionals to join from homes and/ or from their offices (no actual meeting in the conference room took place unless absolutely necessary).

Recent data suggest that SARS‐CoV‐2 can survive on plastic surfaces for up to 72 h;^8^ hence, disinfection with wipes (containing at least 70% of ethanol), and good sanitation practices should be adopted in all physics shared areas. Shared devices such as desktop computer, keyboard, mouse, and phone were wiped as often as possible. Sanitization of all areas including the linac and HDR and console areas was done routinely, however, more stringent sanitizing protocols were already in place to clean all HDR equipments that are in contact with patients.

Housing one physicist on campus and the other working remotely helped sharing the workload while observing all constraints. Tasks such as initial chart check, secondary dose calculations for 3D/VMAT, weekly chart reviews, end‐of‐ treatment chart reviews, etc. were performed remotely. Treatment planning QA for both EBRT and HDR was also done remotely.

Patient‐specific QA tasks were performed after treatment hours to minimize contact with staff and patients. Monthly QAs for the linac were performed by the physicist either over the weekend or late in the afternoon in order to minimize exposure to the pathogens. Sanitizing the work area including the linac and console area was performed after the QA.

Required physical presence during an HDR treatment was practiced by the team where the therapist, “authorized medical physicist,” and “authorized user” stayed at recommended distance away from each other.

On a general note, all patients (visitors and employees) were screened every visit at the front desk with a thermometer and inquired about any symptoms and travel history before they enter the cancer center. If patients presented any symptoms, treatment was discontinued until further notice from the care provider. The physicians were advised to encourage patients with cancer‐not requiring urgent RT‐ to postpone RT at least for three months until the epidemic subsides.

The response to COVID‐19 at the medium‐sized clinic involved testing the entire network stability to make sure that large number of employees can function from home with minimum interruption. Five hundred employees across the school of medicine remotely accessed the network system to perform the operability and stability test, called “pressure test”. The physics team at the central campus decided to divide the physicists and the medical physics residents to two groups: on‐site and off‐site team, which would rotate every two weeks. Each team consisted of three physicists and two medical physics residents. The team members were selected based on their specialties to make sure all special procedures can be covered by each group independently. All team members were equipped with laptops to work remotely. All laptops were prepared by the IT group to support remote access to Eclipse treatment planning system (Varian Medical Systems, Palo Alto, CA) and MOSAIQ record and verify system (MOSAIQ oncology information system, IMPAC Medical Systems, Sunnyvale, CA) through VMware (VMware, Inc. Palo Alto, CA). The computers were also capable of remote desktop to access secondary dose calculation software such as RadCalc® (LifeLine Software, Inc., Austin, TX).

For the purpose of communication, daily huddle meetings were set up through Zoom video conferencing. All team members also communicated through text messages with Tiger Connect (TigerConnect Inc. Los Angeles, CA) application, email, and phone calls. The two on‐site and off‐site teams started their operations on 23 March 2020. The number of patients on external beam treatment did not show a tangible drop due to the COVID‐19 outbreak at the time of reporting (11 April 2020). As the number of RT patients are expected to drop in near future due to increased disease spread, the number of linacs actively treating can be scaled down and the on‐site therapists requirement will be reduced. During the first week of the response, the number of treatment plans doubled as all breast patients on treatment with the active breathing coordinator (ABC) device were re‐planned.^20^ The ROs also introduced few treatment visits and hypofractions where possible. The vital tool that permitted an effective and quick transition to remote working was Mosaiq and Eclipse availability through the cloud. The remote huddle maintained a high level of communication and assisted in solving problems between teams.

The low speed of the internet was a daily challenge for the off‐site team; however the cloud system was able to maintain a reasonable level of speed to function and perform remote tasks. In one instance, a VMware crash resulted in a connection loss and an hour delay for all off‐site team members. Redundancy in remote access was essential for the off‐site team. One important issue was shortage of PPE and masks, one ongoing consideration was to evaluate mask sterilization for re‐use by UV or perhaps ionizing irradiation.

At the large cancer clinic, remote access to treatment planning and treatment management was available via cloud‐based single vendor solution. This provided for sustained operation with almost unlimited licenses for all users in radiation oncology department. After initially sending half of physics and dosimetry staff to remote work‐from‐home on March 16, a week later all of the dosimetry staff were operating from the comfort of their homes. Only a bare‐bone essential physics staff was allowed on‐site in the central location for general and specialized procedures. For SRS and SBRT, Gammaknife, brachytherapy, radiopharmaceutical preparations, proton therapy, adaptive radiotherapy with Halcyon^TM^ and MRIgRT, one individual for each service was mandated. For individual satellite locations, service clusters were formed which provided on‐demand site visits, otherwise all support and review were moved to remote coverage and online audio/video conferencing. Resident rotations were altered to more flexible scenarios where social distancing can be observed and in low‐risk areas of the clinic or done remotely. Graduate students were offered online classes in place of traditional classroom. Treatment planning was reorganized to ensure redundancy of resources, e.g. dynamic jaw tracking feature was disabled to ensure patient transferability across various machines. The initial two weeks saw a rapid increase in treatment plan checks due to replanning to adjust for fractionation change, however it subsided as the number of new patients continued to reduce.

The management in each department considered staffing problems especially should the physicists contract the virus and not able to work. For this purpose, flexibility of the physics team in terms of cross‐coverage was crucial. Training of staff was planned in a way that for each procedure there were several potential back‐up physicists. Detailed instructions were prepared (if not already available) to facilitate training of staff. For example, in the small center, the junior physicist was trained by the senior physicist on all specialized procedures in preparation for if the senior physicist was not able to work. Possibility of recruiting a locum physicist was considered in case when physicist staff shortages appear. In the large center, in order to prepare for a possible shortage of therapists, the dosimetrists who were certified to work as a therapist were identified and the physicists fluent in treatment planning were identified to perform planning if needed.

Table 1, 2 provides a summary of normal approach to physics tasks vs. contingency under the pandemic in all three scenarios.

**Table 2:** Adaptation of various clinical tasks which are performed or assisted by physicists in “Normal scenario” to “Contingency under the pandemic” settings

Task	Normal approach	Contingency under the pandemic
Patient simulation consults	Physical presence if requested for difficult immobilization, etc.	Remote video consultation through video conferencing tools. In some cases it might be possible to use diagnostic images instead of extra CT scans.
Fusion and Registration review	It is only done by the physicists for SBRT or SRS treatments.	Remote registration and fusion reviews.
Initial plan check for EBRT and plan revision check	Perform by a physicist in a shared workspace or individual offices.	Perform remotely from home or in an individual office. Interact through phone/video/email with dosimetrists, therapists, and RO when needed.
On treatment setup verification for SBRT	Physical presence of a physicist for the first SBRT fraction.	Remote review of CBCT and matched images is possible.
Patient‐specific QA for IMRT, VMAT, SBRT, and proton therapy cases	Perform by physics residents, physics assistants, and QA technicians or sometimes by the physicist prior to first fraction. SBRT QA is typically done on the same linac where patient will be treated. Staff are rotated between different satellites for medium to large centers.	Each staff is assigned a machine/clinic to avoid cross‐over. Perform after the completion of all treatments instead of performing during the day. For large centers with multiple satellites, each person (physicist or QA specialist) sticks to his/her machine or satellite. Wait at least 3 hours after treatment. Wear appropriate PPE such as gloves and masks. Preferably, perform alone. If two people were needed, keep 6 feet physical distance. Disinfect equipment after the QA measurements.
General physics consult	Generally to RO, dosimetrist, or therapists in person or on phone. Machine (linacs or proton therapy) calls for trouble shooting.	Done remotely via phone or video conferencing tools. When visiting machine areas, maintain social distancing and wear PPE.
Treatment devices review	Electron blocks and proton compensators and apertures are checked for defects.	Can be accomplished after treatment completion or in a separate location.
*In vivo* dosimetry	Film, ion chamber, TLD, OSLD, diode, MOSFET dosimetry to verify dose to sensitive anatomy or implanted devices.	Physical placement and measurement of dose. In some cases calculations can replace measurement to estimate the dose, e.g. AAPM TG 158.^34^
Machine QA (Daily, monthly, annual, incidental)	Daily: mostly performed by the therapists and reviewed by the physicists remotely. Monthly, annual, and incidental (following a major repair): Performed by residents or physicists.	Daily: remote review, troubleshooting over phone or video devices. Monthly: If under time constraint, limit to essential tests like output verification, and infer other information using trends data from daily QA, e.g. beam profile and imaging. Annual: Can be delayed by few months, or restricted only to essential tests. Incidental: All impacted systems should be tested, however, exercise “social distancing”, disinfect equipment, and wear appropriate PPE when working together.
Brachytherapy planning	Perform by dosimetrist or physicists. Typically near the treatment console with > 5 people around (Physician, physicist, therapist, and trainees).	Planning can be done remotely, with remote reviews and approval, only delivery would mandate physical presence of therapist, authorized user, and authorized medical physicist for HDR.
Radiopharmaceutical Procedures	Y‐90, Lutathera, I‐131 ablation, etc. dose preparation and review by the physicist.	Practice social distancing especially in tightly spaced hotlabs and clean thoroughly after preparation.
Special Procedures: TBI, TSET	Plan and MU calculations are done by physicist; physical presence during TBI fractions.	Plan and MU calculations are done remotely; when attending on linac maintain 6 feet distance and wear appropriate PPE.
Online adaptive treatments	Online adaptive plan development and anatomy of the day review, independent Monte Carlo QA of the plan.	Practice social distancing and wear appropriate PPE when visiting linac for the procedure.
Acceptance and commissioning	Perform by the physicist in the presence of vendor's engineer/team.	Vendor team members are traveling, going through different airports and hotels, so they can potentially be careers of the virus. Perform screening for symptoms. Exercise “social distancing”, disinfect equipment, and wear appropriate PPE when working together.
Weekly chart check	Physicists perform from office.	Physicists perform from office or home. Extra caution must be exercised as some physicians might have decided to change the prescription to a hypo‐fractionation scheme. In some clinics the machines may not be matched (must make sure that if the treatment room has been changed, the machine has had matched dosimetric condition).
Education (residents, graduate students and postdocs, summer interns, undergraduate students, etc.)	On‐site in clinical areas, classrooms, and laboratories.	Online resources. Online lectures are offered through video conferencing tools. Homework submission: online. Exams: Take home. Oral evaluation (or small classes) can be done with individual meetings through internet. Reduced physical presence of laboratories personnel to 20% and only for essential personnel.
Research	On site in class room and laboratories.	Limit to only essential personnel. Prioritize experiments. New students can do literature search in the meantime. If it is absolutely necessary to do experiments for data collection: consider a well‐vented room, wait at least 3 hours after treatment or work over a weekend. Wipe all surfaces and tools (equipment, machine, etc.). Use glove. Exercise social distancing. Do the experiments alone. If two or more persons are needed, use approprate PPE and maintain 6 feet distance between individuals. Data analysis and discussion can be done remotely from home and meeting can be done thorough video/audio conferencing tools.

## 4 DISCUSSION

In the wake of the novel coronavirus pandemic, we presented three models of adaptive scenarios based on the physics manpower employed and treatment workload. These models are particularly relevant to the medical physics workforce in the United States healthcare. Other models, such as public healthcare like in Europe and Canada operate slightly differently and have different staffing for physics related tasks. The current model is more intuitive based on stratification and employment. Since the current model is based on the size of a physics group, it incorporates practice patterns of physics employment in community, academic institution, freestanding cancer clinics, and medical physics group practice. To our knowledge, this work is a one of a kind study on testing scalability of three models under the actual stress of a pandemic. Some of the limitations of this approach include focus on physics workforce, while not considering radiation oncology consultations and referral pattern changes currently taking place. Due to COVID‐19 emergency preparation, some of the ROs may be deployed to primary critical care as urgent response team for the infected individuals, instead of cancer therapy, which would influence their patient numbers and follow ups. Though increase or drop in number of referrals and cancer RT will definitely influence the physics resources, it is too broad a subject to be included in the current manuscript. Similarly, this work was also not aimed at the impacts or scalability of diagnostic radiological physics tasks in radiography or diagnostic CT, MRI, PET, ultrasounds, and other portable imaging or therapy equipment and scanners. The radiology departments, in general, tend to have few in‐house physics staff, most of their quality assurance needs are served through third party physics services or vendors.

The clinical devices such as linacs, high dose‐rate brachytherapy afterloaders, Gamma Knife, tomotherapy, CyberKnife, and proton therapy equipment and devices, require specialized preventive maintenance and inspections. Service engineers specifically trained on these devices present another weak link due to their small number, where a combination of staff illness and equipment failure or unavailability of spare‐parts and/travel restrictions can result in cessation of radiotherapy services in a small clinic.

The remote working practice suffers from lack of interpersonal communication, frequent internet disconnections and slowness, and malfunction of the software. In small or large cloud‐based systems, glitches do happen, due to large data transfers, bottle necks in the data pipelines, slow data transfer rates and connection drops, which can lead to outage of all RT services across all connected facilities. A back‐up connection or alternative routing can allow for continuity of services in some cases.

Although all three models appear to be successfully implemented in the face of all challenges at this point in time, it would be worth investigating their perseverance during the full peak of the disease. Flexibility of personnel when needed in covering different tasks is critical to ensure smooth operation. In large centers, the physics staffing levels may include individuals who have been out of clinical practice for some period. Written procedures, documentation, and training videos can provide some solution to bring them up to speed. Of important note is the development and implementation of risk‐assessment techniques and failure mode and effects analysis (FMEA)^32^ in disruptive situations. FMEA and criticality analyses must be performed in scenarios of scale up or scaling down operations to envisage the limitations of each contingency plan, especially in deciding what QA tests are more vital than others. Issues related to education and research were not further discussed in detail in this report in the interest of space and continuity of clinical operation.

This work sheds some light on adaptability of clinical physics practice under the real life stress test. The clinical services in three model scenarios are able to adapt to the current level of disease spread in their local background. The corporeal presence of clinical physics personnel has reduced significantly in various parts of cancer clinics limiting only to essential personnel, without significantly impacting the service at this point. However, this requires continuous prioritization of only essential and urgent tasks by following the time, distance, and shielding principle of radiation protection, when physics presence is absolutely unavoidable.

## 5 CONCLUSION

Clinical medical physicists play a vital role in ensuring safe and effective delivery of radiotherapy in a cancer clinic. Under disruption from COVID‐19 pandemic, some aspects of the approach to medical physics tasks have rapidly changed. Overall, the approach has been to adopt a “dirty” team and a “clean” team approach in all three models. For a small clinic model, with scarce resources a smooth operation can be established with availability of remotely accessible software, at this point in disease spread. Scope of physics services need to be modified to meet the clinical objectives; medium and large hospitals benefit from using standardized tools and large staff pools who are able to takeover in case of a major disease related staff illness.

## CONFLICTS OF INTEREST

The authors have no conflict of interest to disclose.
